# Genome-wide identification and comprehensive analysis of the *COI1* gene family in *Medicago sativa*

**DOI:** 10.3389/fpls.2025.1712214

**Published:** 2025-11-19

**Authors:** Fangqi Chen, Luran Wang, Ruifang Jia, Jun Zhao, Kejian Lin, Qing Zhao, Zhengqiang Chen, Yuanyuan Zhang

**Affiliations:** 1 Key Laboratory of Biohazard Monitoring, Green Prevention and Control for Artificial Grassland, Ministry of Agriculture and Rural Affairs, Institute of Grassland Research of Chinese Academy of Agricultural Sciences, Hohhot, China; 2 College of Horticulture and Plant Protection, Inner Mongolia Agricultural University, Hohhot, China

**Keywords:** COI1, *Medicago sativa* L., phylogenetics, cis-regulatory elements, AMV

## Abstract

COI1 (CORONATINE INSENSITIVE) is a central regulator of plant defense against biotic stresses, yet its family members remain uncharacterized in the globally important forage crop alfalfa (*Medicago sativa* L.). Here, we identified 32 *MsCOI1* genes, which were phylogenetically classified into four subfamilies (I–IV). The four subfamilies each contain 5, 6, 9, and 12 *MsCOI1* genes, respectively. Structural analysis revealed conserved three-exon architecture and six critical motifs, with Motif 3 exclusively absent in Subfamily IV. Promoter cis-element profiling showed enrichment in stress-responsive elements: 93.8% contained ABRE (abscisic acid response), 68.8% harbored MeJA-responsive elements, and 50% possessed TC-rich repeats (defense/stress response). Expression dynamics demonstrated tissue specificity (e.g., *MsCOI1-24/25/26* in nodules/roots) and hormone responsiveness (40.6% induced by MeJA/ABA). Crucially, six of eight tested *MsCOI1s* were upregulated during *Alfamovirus* AMV (commonly known as Alfalfa mosaic virus, AMV) infection. However, *MsCOI1–8* was significantly inhibited at 5 dpi, indicating that the virus manipulates the COI1-mediated plant response. This study provides the first comprehensive resource of *MsCOI1* genes for enhancing viral resistance in alfalfa breeding.

## 1 Introduction

Jasmonates (JAs), a class of oxylipin derived phytohormones, orchestrate multifaceted physiological processes spanning growth modulation, floral transition, senescence, and responses to biotic/abiotic stressors (Li et al., 2021). This pathway initiates with JA conjugation to isoleucine (JA-Ile) by JAR1, enabling its recognition by the F-box protein coronatine insensitive 1 (COI1) (Yan et al., 2009). The core nucleocytoplasmic JA signaling module comprises the SCF^COI1^ E3 ubiquitin ligase complex, JAZ repressors, and MYC2 transcription factors, collectively termed the COI1-JAZ-MYC2 regulatory axis (Jose et al., 2008). Upon pathogens challenge, JA is transformed into its active form, the isoleucine conjugate JA-ILE (Liao et al., 2022). After the downstream receptor COI1 of jasmonic acid recognizes JA-ILE, it targets the JAZ inhibitor and degrades it through the 26S proteasome (Wasternack and Hause, 2013; Howe et al., 2018), and then release MYC2 transcription factor to activate JA-responsive genes (such as protease inhibitors, secondary metabolite synthesis genes) and stress adaptation mechanisms, thereby activating the expression of pathway related disease-resistant genes (Gareth, 2020). Notably, the JA pathway exhibits context dependent duality in plant virus interactions: it may serve as a positive regulator of antiviral resistance or be subverted by viral effectors to enhance host susceptibility (Yan and Xie, 2015). Overexpression of *SlJAZ7* in tomato (*Solanum lycopersicum*) mitigates reactive oxygen species (ROS) accumulation and bolsters disease resistance (Lin et al., 2024). Exogenous JA application induces systemic acquired resistance (SAR) in *Arabidopsis thaliana* against viral infections by upregulating pathogenesis-related (PR) genes (Steven et al., 2003). In contrast, the *Cucumovirus* CMV (commonly known as Cucumber mosaic virus, CMV) 2b protein, an RNA-silencing suppressor, directly dysregulates JA signaling to potentiate virulence (Wu et al., 2017). JA signaling may compromise resistance against *Tobamovirus tabaci* (commonly known as Tobacco mosaic virus, TMV) by antagonizing salicylic acid-dependent defenses (Oka et al., 2013).

COI1, identified as the canonical JA receptor (Yan et al., 2009), is also an important component of the core module of jasmonic acid signal transduction (Sheard et al., 2010). Evolutionary analyses reveal lineage-specific expansion via whole-genome duplications (WGDs) and tandem repeats, with COI1 copies exhibiting functional divergence (Duan et al., 2016). COI1 belongs to a multigene family and encodes F-box/LRR proteins essential for JA perception. Proteins containing F-box domains are integral components of Skp/Cullin/F-box (SCF)-type E3 ubiquitin ligase complexes, conferring substrate specificity. Mutations in other components or regulators of the SCF complex, such as AXR1, CUL1, RBX, and JAI4/SGT1b, also exhibit JA insensitivity, demonstrating the critical role of COI1 in the JA pathway (Chini et al., 2009). Beyond its roles in developmental processes (e.g., senescence, flowering, male fertility), COI1 governs defense responses against insect herbivory and microbial pathogens. In *Arabidopsis*, *AtCOI1* null mutants display male sterility and heightened susceptibility to pathogens (Feys et al., 1994), while rice *OsCOI1b* knockout delays leaf senescence but compromises grain filling (Lee et al., 2015). Recent studies highlight COI1 paralogs in crops like wheat (*TaCOI1*) and maize (*ZmCOI1*) as key regulators of defense-gene networks. Silencing *TaCOI1* increases powdery mildew (*Blumeria graminis*) penetration by 40% (Jing et al., 2018), and the overexpression of *ZmCOI1a* restores fungal resistance in *Arabidopsis coi1* mutants (An et al., 2019). These findings underscore COI1’s dual role in development and stress adaptation, driven by structural variations in F-box/JAZ-binding domains (Sheard et al., 2010). Despite advancements, systematic characterization of COI1 families in polyploid legumes, particularly alfalfa, remains unexplored.

Alfalfa (*Medicago sativa* L.), a globally cultivated forage legume, is renowned for its high protein content (18-25%), essential minerals, and vitamins, earning it the title ‘King of Forages’ (Chen S. et al., 2020). With over 2,000 years of cultivation history in China, it made China the world’s second largest producer (Guo et al., 2022). It supports livestock nutrition and enhances soil fertility through nitrogen fixation, playing a critical role in sustainable agriculture (Wang et al., 2003). However, alfalfa production faces severe threats from *Alfamovirus* AMV (commonly known as Alfalfa mosaic virus, AMV). AMV first reported in U.S. alfalfa in 1931, is a highly destructive plant pathogen capable of infecting numerous important economic crops, causing symptoms such as mosaic, leaf crinkling, and even plant death (Bos, 1971). Field surveys indicate that 94% of Chinese alfalfa cultivars are susceptible to AMV, with only 3.3% (e.g., ‘Archer 2’) showing strong resistance (Guo et al., 2019). Traditional control relying on insecticides against aphid vectors is increasingly ineffective due to pesticide resistance, highlighting the need for genetic solutions.

As a autotetraploid plant, the complexity of alfalfa’s genome (e.g., gene redundancy, subgenome differentiation) has added many difficulties to the study of gene families. The expansion pattern and functional redundancy mechanism of the COI1 family in polyploid genomes remain unclear. This study aims to systematically investigate the *COI1* gene family in alfalfa. We identified a total of 32 *MsCOI1* genes and conducted comprehensive analyses of their physicochemical properties, chromosomal localization, phylogenetic relationships, gene structure, conserved protein domains, cis-acting regulatory elements, and expression patterns. By characterizing the sequence features and expression profiles of the *MsCOI1* gene family, this research provides valuable candidate gene resources for molecular breeding aimed at enhancing viral resistance in alfalfa.

## 2 Materials and methods

### 2.1 Plant materials

Seeds of alfalfa ‘Zhongmu No.1’ were commercially procured from Gansu Jiuquan Daye Seed Co., Ltd. The AMV infectious inoculum, originally field collected from Chifeng, Inner Mongolia, was maintained under cryopreservation at -80 °C in laboratory repository. Plants were established in controlled environment chambers under 16-h photoperiod/8-h dark regime with diurnal temperature modulation (25 °C day/22 °C night). Root, stem, nodule, leaf, and floral tissues were dissected from four phenotypically uniform ‘Zhongmu No.1’ plants at the fourth-trifoliate stage. Foliar spraying with 100μmol/L (Duan et al., 2016; Ma et al., 2023) aqueous solutions of abscisic acid (ABA) and methyl jasmonate (MeJA) was administered to fourth trifoliate stage plants. Treated specimens were maintained with 16-h light/8-dark cycles. Leaf samples were collected at sequential timepoints (0 and 24 h post treatment) for transcriptional analysis. At the fourth trifoliate stage, AMV infection was induced via mechanical rub-inoculation using the preserved inoculum. To elucidate the early temporal dynamics of AMV infection, systemic leaves from inoculated and mock-treated control plants were harvested at 3, 5, and 7 dpi and immediately flash frozen in liquid nitrogen for downstream analyses.

### 2.2 RNA extraction and first-strand cDNA synthesis

Three biological replicates were collected per sample and immediately flash frozen in liquid nitrogen. Total RNA was isolated from plant leaves using the TransZol Up Plus RNA Kit (TransGen Biotech, Beijing, China; Cat# ER501). All cDNA used as templates for cloning was synthesized by reverse transcription of 1.0 μg RNA extracted from leaves of alfalfa ‘Zhongmu No.1’ using the HiScript II Q RT SuperMix for qPCR (Vazyme, Nanjing, China; Cat# R223) and subsequently employed for RT-qPCR analysis.

### 2.3 Genome-wide identification of *MsCOI1* genes

The reference amino acid sequences of the seven *Arabidopsis* AtCOI1 were retrieved from the TAIR (https://www.arabidopsis.org/). Homology-based screening was performed against the tetraploid alfalfa ‘Xinjiang Daye’ genome assembly (https://figshare.com/projects/whole_genome_sequencing_and_assembly_of_Medicago_sativa/66380) to provisionally identify *MsCOI1* family members. Candidate sequences underwent rigorous validation using the SGS domain (PF05002) profile from the Pfam database (http://www.ebi.ac.uk/interpro/entry/pfam/PF05002/) and conserved domain architecture analysis via CDD (http://www.ncbi.nlm.nih.gov/Structure/cdd).

### 2.4 Phylogenetic reconstruction of *MsCOI1*

Thirteen *Zea mays* and seven *Oryza sativa* COI1 protein sequences were acquired from Phytozome v13 (https://phytozome-next.jgi.doe.gov/). Seven *Arabidopsis* COI1 protein sequences were obtained from TAIR (https://www.arabidopsis.org/). A phylogenetic tree was constructed with MEGA 11.0 using the NJ (Neighbor-Joining) algorithm incorporating alfalfa, *Arabidopsis*, maize, and rice homologs (**Supplementary Table 1**). Topological robustness was assessed with 1,000 bootstrap replicates.

### 2.5 Computational prediction of MsCOI1 protein properties

Physicochemical attributes (amino acid count, molecular weight, theoretical pI, GRAVY index) were computed using ExPASy ProtParam (https://prosite.expasy.org/protparam). Subcellular localization was inferred via CSBIO (http://www.csbio.sjtu.edu.cn/bioinf/euk-multi-2/).

### 2.6 Chromosomal mapping of *MsCOI1* genes

Genomic coordinates of *MsCOI1* genes were extracted from the ‘Xinjiang Daye’ annotation files. Physical positioning and visualization were conducted with TBtools using chromosome-scale scaffolds.

### 2.7 Consensus motif analysis of *MsCOI1* proteins

Conserving motifs were investigated using the MEME online tool (Version 5.4.1) (http://meme-suite.org/) with the following parameters: maximum motif count = 6, minimum width = 6 amino acids, maximum width = 50 amino acids. Resultant motif architectures were visualized in TBtools.

### 2.8 Structural annotation of *MsCOI1* genes

Exon-intron organizations were resolved by aligning *MsCOI1* genomic sequences with corresponding GFF3 annotations. Schematic representations were generated using TBtools’ Gene Structure Viewer.

### 2.9 *In silico* promoter cis-element screening

The 2,000 bp upstream regulatory regions were excised using TBtools. Putative cis-regulatory elements were annotated via PlantCARE (https://bioinformatics.psb.ugent.be/webtools/plantcare/html/) and graphically summarized in TBtools.

### 2.10 Expression profiling of *MsCOI1* genes

Transcripts harboring defense/stress responsive cis-elements were selected for RT-qPCR validation. Gene specific primers were designed in Premier 6.0 and commercially synthesized by Qingke Biotechnology (Beijing, China). Amplifications were performed using ChamQ Blue SYBR qPCR Master Mix (Vazyme Biotech, Q312-02) on a QuantStudio 5 PCR system (Thermo, Waltham, MA, USA). Experiments were performed with three replicates and transcript abundance was quantified via the 2^−ΔΔCT^ method with *MsActin* as the endogenous control. RT-qPCR primers are shown in **Supplementary Table 7**.

### 2.11 RNA-seq analysis

RNA-seq analysis was performed by Genepioneer Biotechnologies (Nanjing, China). Total RNA was assessed for integrity using 1% agarose gels, purity with NanoPhotometer^®^ spectrophotometer (IMPLEN, USA), concentration via Qubit^®^ 2.0 Fluorometer (Life Technologies, USA), and integrity number (RIN) with Agilent Bioanalyzer 2100 (Agilent Technologies, USA). Libraries were constructed from 3 μg RNA/sample using NEBNext^®^ Ultra™ RNA Library Prep Kit (NEB, USA), followed by fragmentation, cDNA synthesis (M-MuLV Reverse Transcriptase), end repair, adapter ligation, and size selection (AMPure XP, Beck-man Coulter, USA). Libraries were clustered on cBot (Illumina, USA) and sequenced (150 bp paired-end) on Illumina NovaSeq 6000. Raw reads were quality controlled (in-house Perl scripts), removing adapters/poly-N/low-quality sequences. Clean reads were aligned to the reference genome using HISAT2. Transcript assembly and FPKM quantification used StringTie. Differential expression analysis employed DESeq (v1.10.) for replicated samples (FDR<0.05) or edgeR for non-replicated samples (FDR<0.01, |log2FC|≥1). Functional annotation utilized Nr, Nt, Pfam, KOG, Swiss-Prot, KEGG KO, and GO databases; enrichment analyses used GO seq and KOBAS (v2.0.).

### 2.12 Transcriptome data collection and analysis

The transcriptome data corresponding to the *MsCOI1* genes in different tissues of alfalfa plants were obtained from the NCBI short read archive database (www.ncbi.nlm.nih.gov/sra/) as accession SRP055547 (O'Rourke et al., 2015). Gene expression levels were calculated based on Fragments Per Kilobase of exon model per Million mapped fragments (FPKM) values. Differentially expressed genes (DEGs) were subsequently identified and used to generate heatmaps, with visualization performed using TBtools.

## 3 Results

### 3.1 Alfalfa *MsCOI1* genes identification and characterization

Based on the conserved amino acid sequences of the *Arabidopsis* AtCOI1 proteins, the SGS domain (PF05002) profile from the Hidden Markov Model (HMM), and the Conserved Domains Database (CDD) database, a total of 32 *MsCOI1* genes were identified within the genome of the autotetraploid alfalfa cultivar ‘Xinjiang Daye’. Analysis of their physicochemical properties revealed that the proteins encoded by the *MsCOI1* genes range in length from 506 (*MsCOI1-20*) to 616 (*MsCOI1-18*) amino acids. Their predicted molecular weights span from 57.67 kDa (*MsCOI1-24, MsCOI1–25* and *MsCOI1-26*) to 68.97 kDa (*MsCOI1-18*), and their theoretical isoelectric points (pI) range between 5.12 (*MsCOI1-13*) and 9.02 (*MsCOI1-20*). The average grand hydropathy (GRAVY) values for all 32 MsCOI1 proteins were negative, indicating overall hydrophilic character (**Supplementary Table 3**). Subcellular localization prediction of the 32 MsCOI1 proteins revealed that 22 of them were exclusively localized to the nucleus (**Supplementary Table 3**). The remaining 10 proteins were distributed as follows: five (MsCOI1-7/9/18/30/31) were localized in the cytoplasm, and the other five (MsCOI1-5/11/12/13/15) were found to be localized in both the nucleus and cytoplasm. The MsCOI1 proteins were mainly constructed based on Alpha helix and random coils, and most of the MsCOI1 proteins had around 50% distribution of α-helices (**Supplementary Table 4**).

### 3.2 Chromosomal localization analysis of the *MsCOI1* gene family

**Figure 1:**
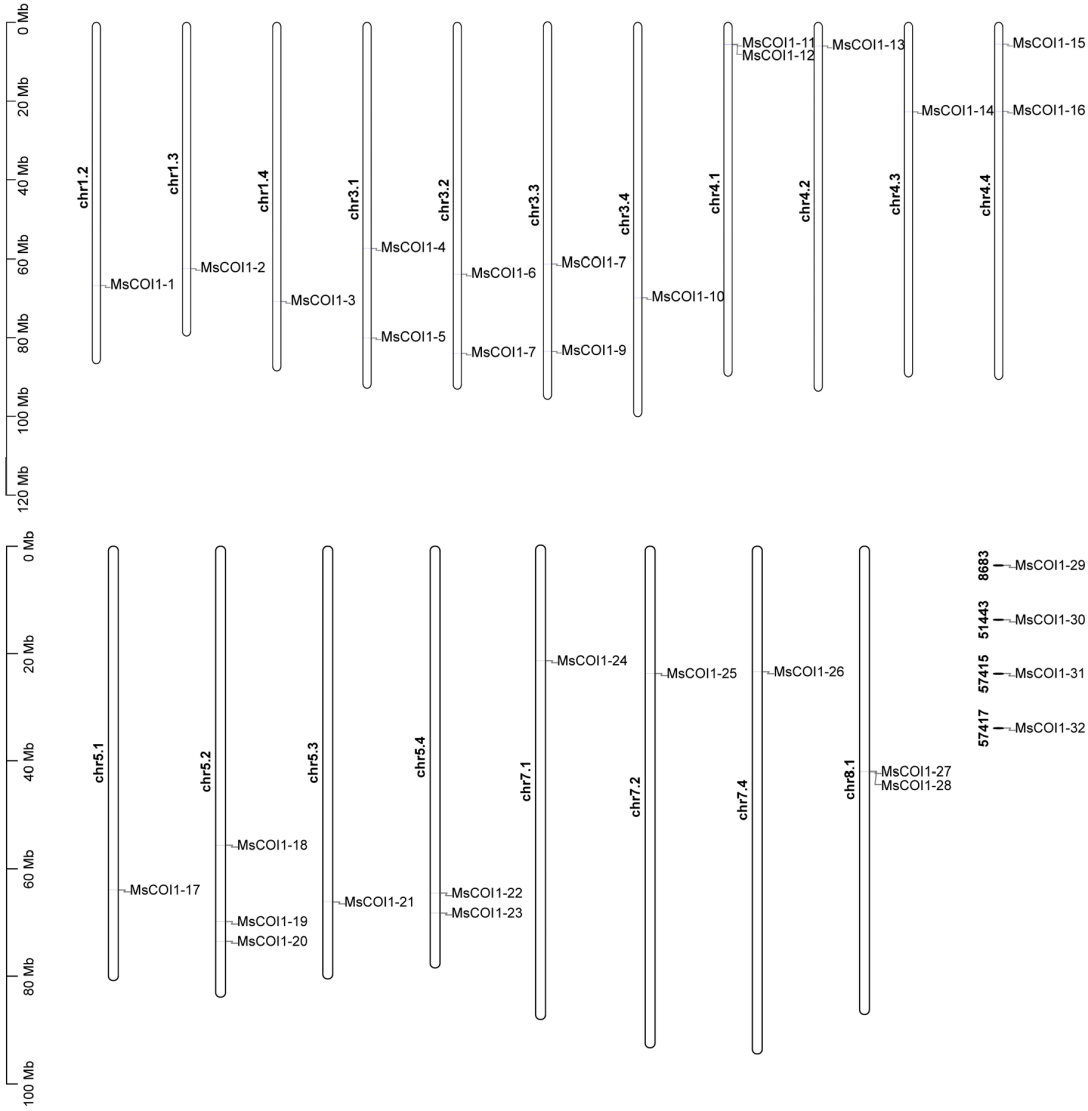
Chromosomal location of the identified *MsCOI1* genes on alfalfa chromosomes. The chromosomal localization of *MsCOI1* genes were determined based on the XinJiangDaYe genome annotation files.

Based on the annotation information of the alfalfa genome, 28 out of the 32 identified *MsCOI1* genes were localized to 19 chromosomes (**Figure 1**). Among these, chromosomes chr1.2, chr1.3, chr1.4, chr3.4, chr4.2, chr4.3, chr5.1, chr5.3, chr7.1, chr7.2, and chr7.4 each harbor a single *MsCOI1* gene. Chromosomes chr3.1, chr3.2, chr3.3, chr4.1, chr4.4, chr5.4, and chr8.1 each contain two *MsCOI1* genes. Chromosome chr5.2 contains three *MsCOI1* genes. Furthermore, one *MsCOI*1 gene was identified on each of the unassembled genomic scaffolds 8683, 51443, 57415, and 57417. In particular, as we observe in **Figure 1**, there are two closely arranged *MsCOI1* genes on both chromosome chr4.1 and chr8.1, and they may be a small tandem repeat cluster. **Supplementary Table 2** shows the names, IDs, and amino acid sequences of these genes.

### 3.3 Phylogenetic analysis of the *MsCOI1* gene family

**Figure 2:**
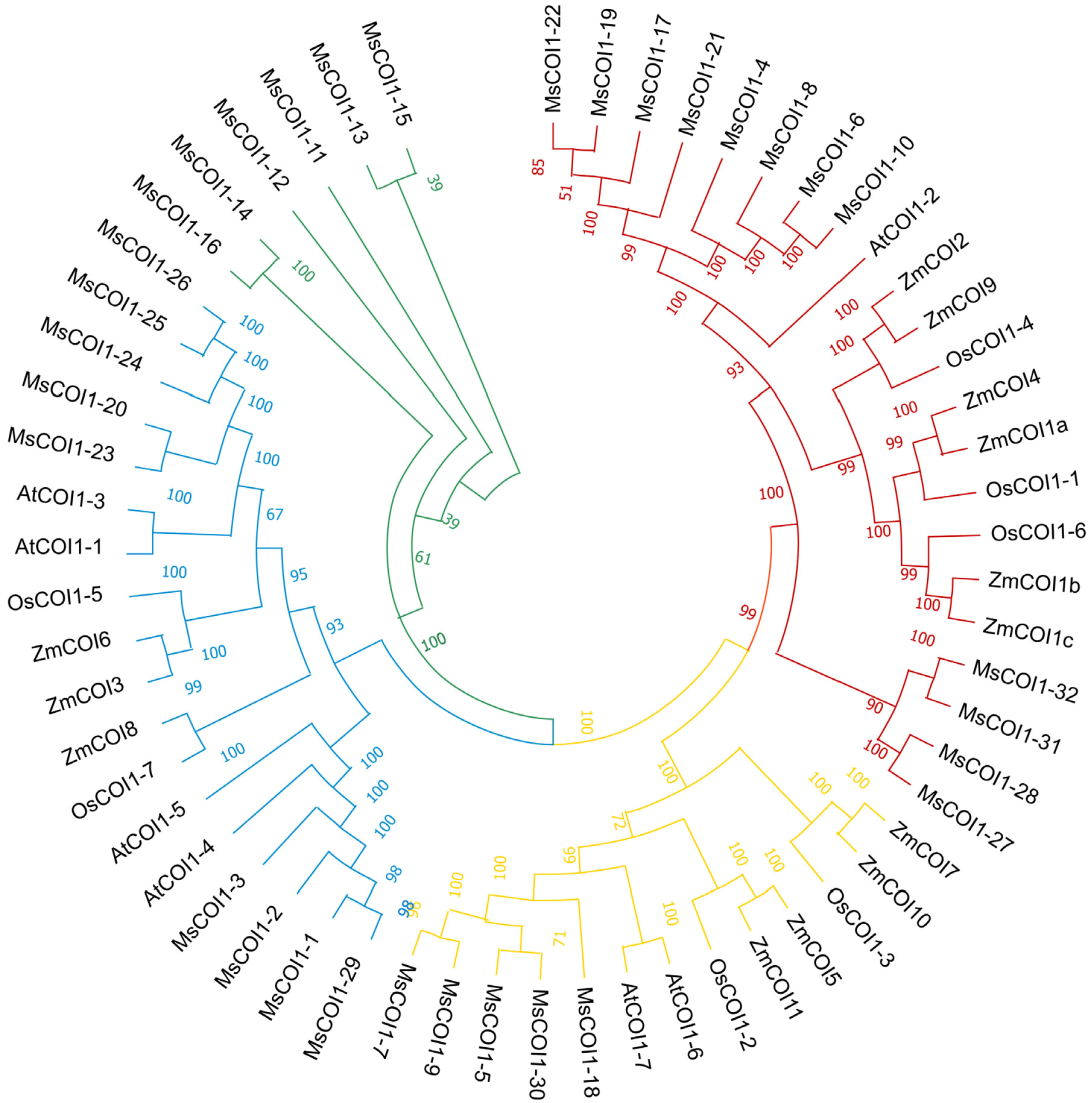
Phylogenetic tree of MsCOI1 proteins from alfalfa, maize, rice, and *Arabidopsis*. The phylogenetic tree was constructed using COI1 amino acid sequences by the neighbor-joining (NJ) method with 1,000 bootstrap replicates. The phylogenetic tree was divided into four groups shown in different colors. The bootstrap support values >75% were shown.

To investigate the evolutionary relationships between the *MsCOI1* gene family and its orthologs in representative plant species, a comprehensive phylogenetic analysis was conducted. Full length amino acid sequences of 56 COI1 proteins from four taxa (alfalfa, maize, rice, and *Arabidopsis*) were aligned using ClustalW (default parameters). An unrooted phylogenetic tree was constructed via the Neighbor-Joining (NJ) method in MEGA11.0 software (Version 11.0.13), with pairwise deletion for gap treatment. The resulting phylogenetic topology revealed clear clustering patterns (**Figure 2**). Thirty-two MsCOI1 proteins were categorized into four distinct subfamilies with strong statistical support (bootstrap values >75%). Subfamily I contained 12 MsCOI1 members, clustering closely with AtCOI1-2, but not closely with monocot-specific COI1 orthologs such as ZmCOI1 and OsCOI1. Subfamily II comprised five MsCOI1 proteins, exhibiting high homology to stress-responsive dicot proteins including AtCOI1–6 or AtCOI1–7 from *Arabidopsis*. Subfamily III included nine MsCOI1 members, forming a divergent clade with OsCOI1 and ZmCOI1. Subfamily IV consisted of six MsCOI1 members, showing a separate branch with only the alfalfa gene.

### 3.4 Gene structure and conserved domain analysis of the *MsCOI1* family

**Figure 3:**
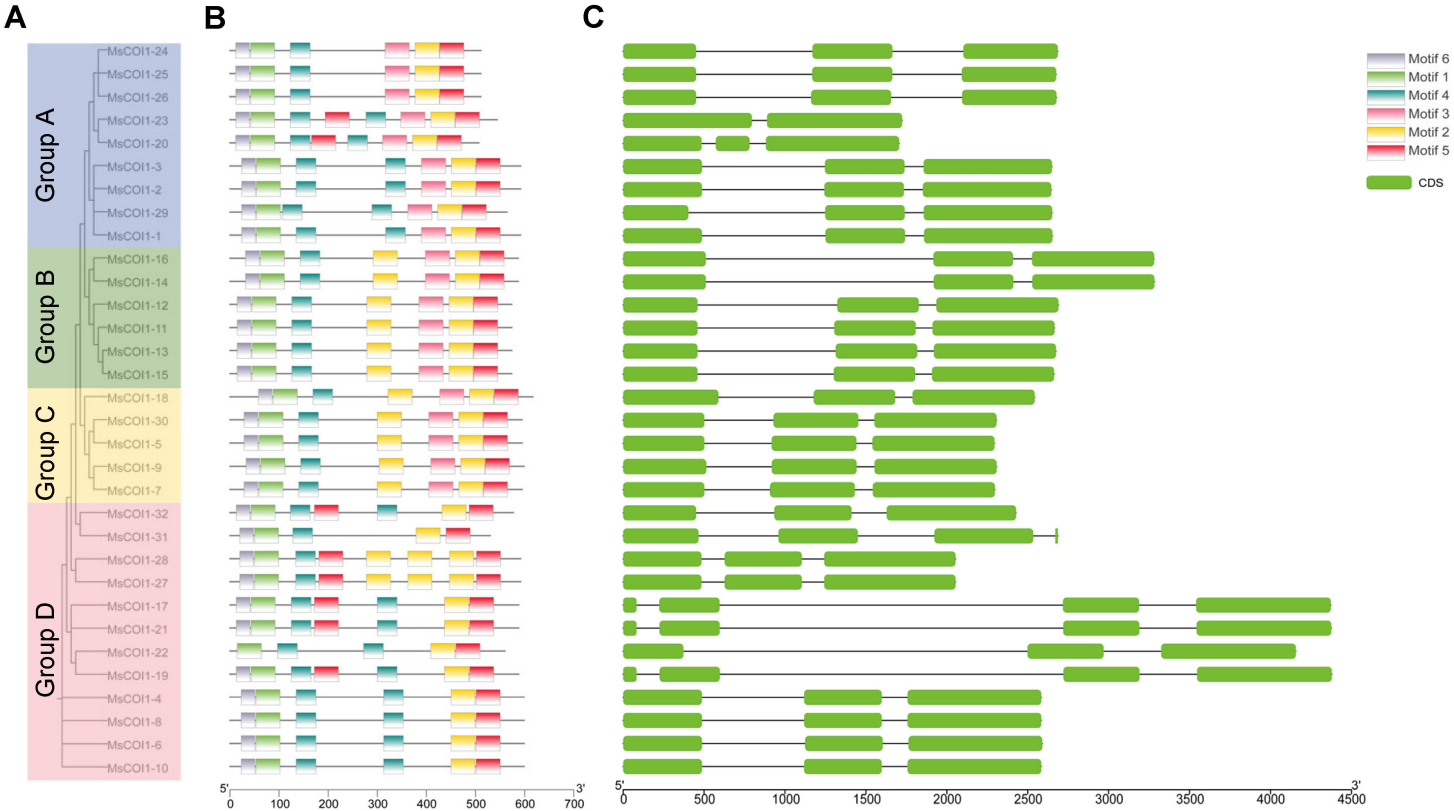
Analysis of conserved motifs and gene structure in the MsCOI1 genes. **(A)** The phylogenetic tree of MsCOI1 protein in alfalfa. The phylogenetic tree of the COI1 amino acid sequence was constructed by using the neighbor-joining (NJ) method, and 1000 bootstrap repetitions were carried out; **(B)** Six types of conserved motifs were predicted in the MsCOI1 protein sequences. The different motifs were shown in different color boxes; **(C)** CDS regions and introns were shown as green boxes and horizontal lines, respectively.

To characterize the structural organization of *MsCOI1* genes, we systematically analyzed their intron-exon architectures. Genomic sequences and corresponding coding sequences (CDS) of all 32 *MsCOI1* genes were aligned to determine exon-intron boundaries. The analysis revealed that the majority of *MsCOI1* genes (27 out of 32, representing 84.4%) exhibited a conserved three-exon structure. Among the remaining genes, one *MsCOI1* member (1 out of 32, 3.1%) contained two exons, while four genes (4 out of 32, 12.5%) possessed four exons (**Figure 3C**). This predominant conservation of exon number indicates evolutionary constraint on gene structure within the *MsCOI1* family.

Six distinct conserved motifs (Motif 1–Motif 6) were identified across the 32 *MsCOI1* protein sequences using the MEME online tool (**Figure 3B**). Four motifs, Motif1, Motif2, Motif4 and Motif5, exhibited absolute conservation, being universally present in all 32 MsCOI1 proteins. Motif 6 showed near-universal conservation, detected in 31 MsCOI1 proteins with the exception of MsCOI1-22. Subfamily-specific motif distribution patterns were identified. Motif 3 was universally conserved across all members of Subfamilies I and II, whereas it was entirely absent from Subfamily III members (**Figures 3A, B**). This differential motif architecture suggests functional divergence between subfamilies, particularly in signaling complex assembly.

### 3.5 Analysis of cis-regulatory elements in the promoter regions of *MsCOI1* genes

**Figure 4:**
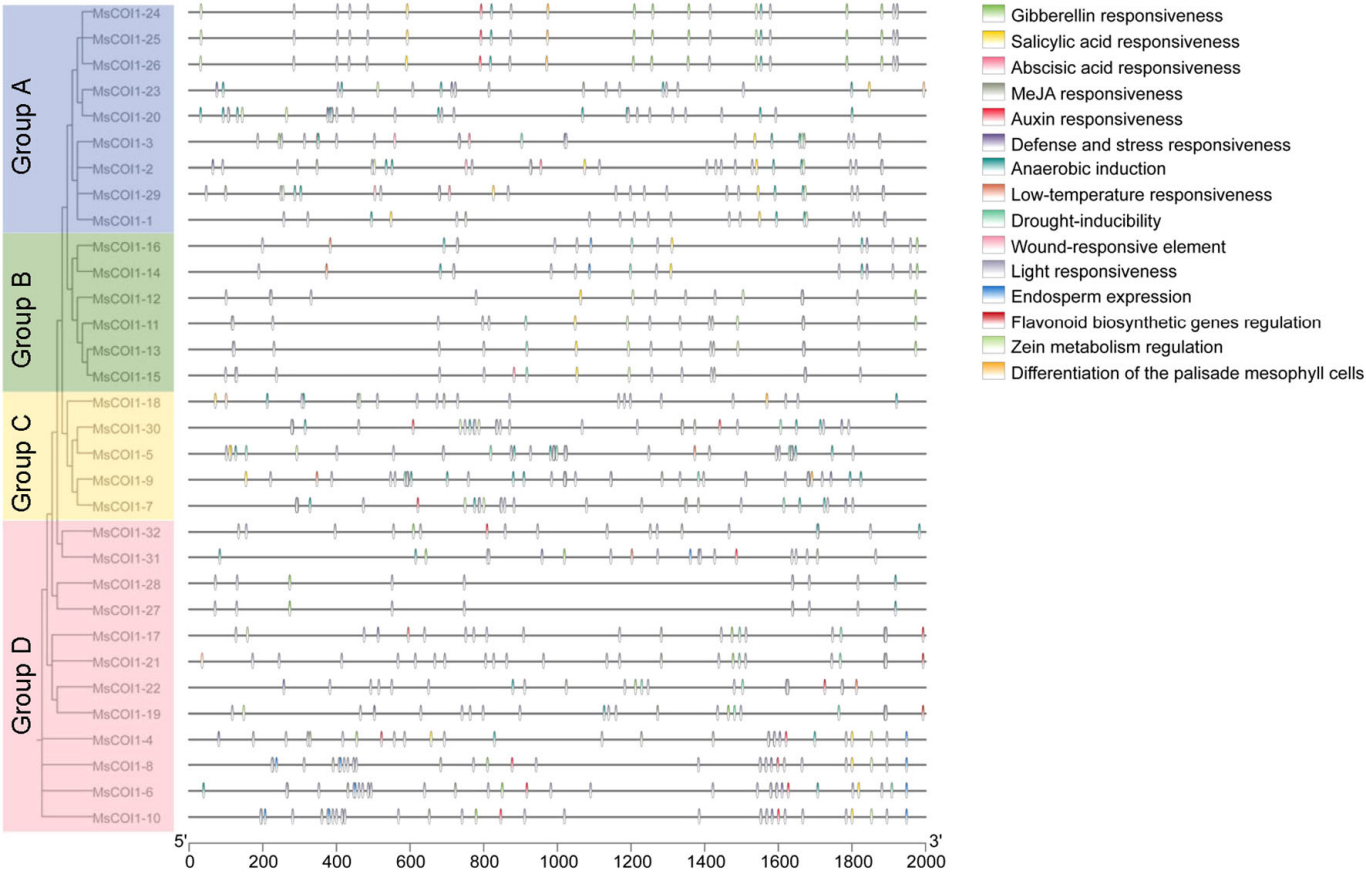
Prediction of cis-acting elements in the *MsCOI1s* promoter regions.

To investigate potential cis-elements involved in transcriptional regulation of the *MsCOI1* gene family, we performed comprehensive prediction analysis on the 2000 bp promoter sequences upstream of the translation start sites for all *MsCOI1* genes. This analysis identified five categories of plant hormone responsive elements and five categories of stress responsive elements within the promoter regions (**Figure 4**). All 32 *MsCOI1* genes contained at least one plant hormone responsive element. Among these, abscisic acid responsive elements (ABRE) demonstrated the highest ubiquity, being present in 30 out of 32 *MsCOI1* promoters (93.8%), with the exception of *MsCOI1–1* and *MsCOI1-18*. Furthermore, methyl jasmonate responsive elements (CGTCA-motif/TGACG-motif) were detected in the promoter regions of 22 *MsCOI1* genes (68.8%), and gibberellin-responsive elements (GARE-motif/P-box) and salicylic acid responsive elements (TCA-element) were each identified in 21 *MsCOI1* promoters (65.6%). Auxin responsive elements (AuxRE/TGA-element) exhibited the lowest frequency, occurring in only 11 *MsCOI1* promoters (34.4%). Regarding stress-responsive elements, defense and stress responsive elements (TC-rich repeats) were the most abundant category, present in the promoters of 16 *MsCOI1* genes (50.0%), followed by drought-inducibility elements (MBS) in 14 promoters (43.8%). Low-temperature responsiveness elements (LTR) were found in eight *MsCOI1* promoters (25.0%), while wound responsive elements (WUN-motif) and anaerobic induction elements (ARE) showed minimal occurrence, being present in only three (9.4%) and two (6.3%) *MsCOI1* promoters respectively. The joint analysis of the action elements and the four subfamilies of system evolution analysis (as shown in **Figure 4**) revealed that there were more anaerobic induction related elements in the *MsCOI1* genes of group A. The *MsCOI1* genes in group B annotated fewer cis-acting elements than the other subfamilies, but salicylic acid and gibberellin response elements were present in all of them. Each gene in Group C was labeled with MeJA response elements. The *MsCOI1* genes in group D has multiple endosperm expressions and auxin responsiveness elements. The differences in cis-acting elements among these subfamilies may suggest differences in their gene functions.

### 3.6 Tissue-specific expression patterns of *MsCOI1* genes

**Figure 5:**
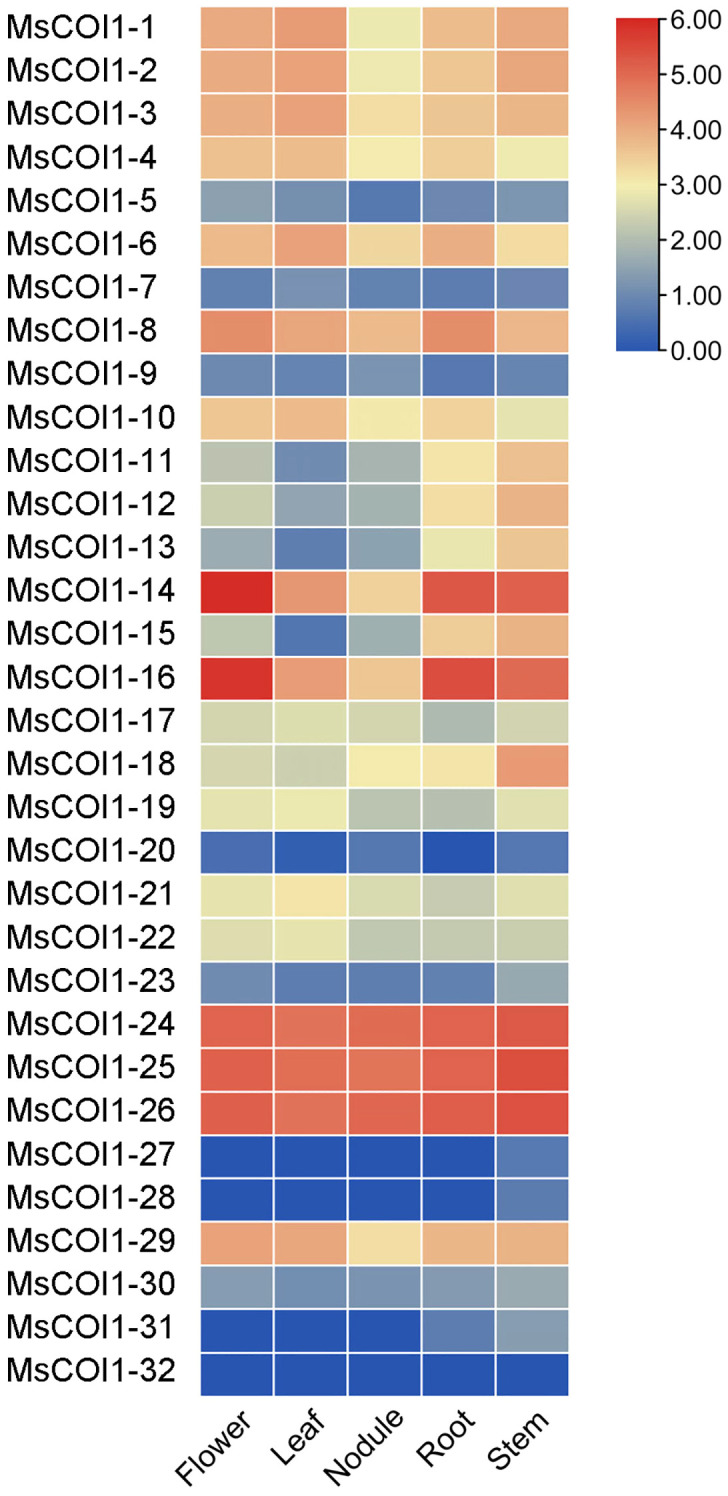
Expression levels of *MsCOI1s* in different tissues. Transcriptome data were used to analyze the samples of roots, nodule, stems, leaves, and flowers. The gene expression level was calculated based on the FPKM value. Red and blue represent high and low expression levels, respectively. The raw are provided in **Supplementary Table 8**.

To determine the expression profiles of *MsCOI1* family genes across different alfalfa tissues, we analyzed their transcript abundance in five distinct organs (stems, nodules, roots, leaves, and flowers) based on transcriptome data from the NCBI short read archive database(www.ncbi.nlm.nih.gov/sra/). All 32 *MsCOI1* genes exhibited detectable expression in at least one of the five examined tissues, with significant variation in transcript levels observed among different organs (**Figure 5**). Specifically, seven genes (*MsCOI1-5/7/9/20/27/28/32*) showed consistently low expression levels across all tissues. In contrast, three genes (*MsCOI1-24/25/26*) demonstrated constitutively high expression in all five tissues, with the highest abundance observed in nodules and roots.

### 3.7 Expression patterns of *MsCOI1* genes under exogenous hormone treatments

**Figure 6:**
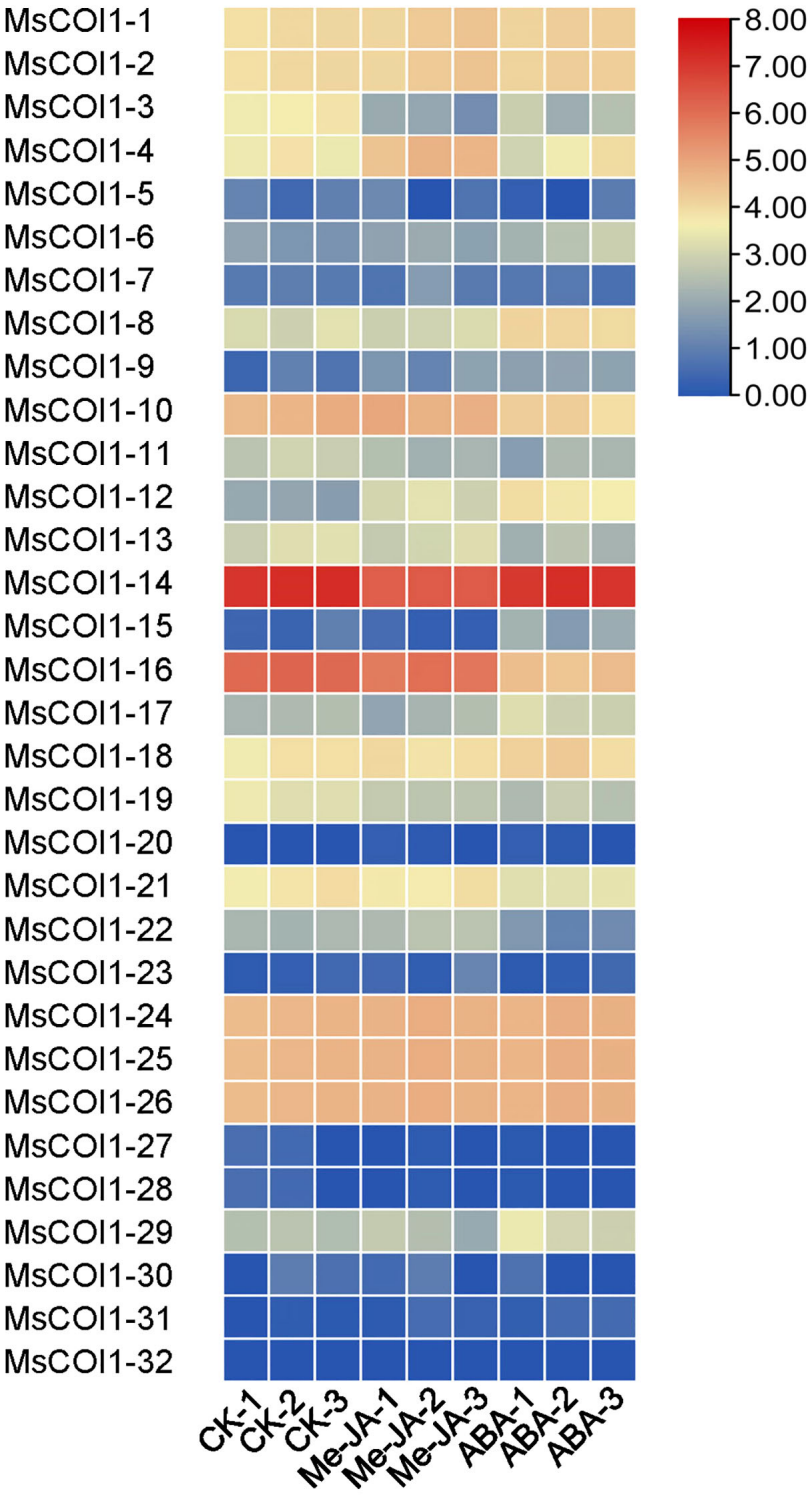
The expression profiles of 32 *MsCOI1* genes under 100 mM exogenous methyl jasmonate (MeJA) and abscisic acid (ABA) spraying treatments. The data represented three independent biological replicates. Gene expression levels, quantified using FPKM values, were depicted with red and blue indicating high and low expression, respectively. Raw data were available in **Supplementary Table 9**.

To investigate the transcriptional responses of all 32 *MsCOI1* family genes to phytohormone elicitation, we analyzed their expression profiles under exogenous methyl jasmonate (MeJA) and abscisic acid (ABA) spraying treatments using transcriptome sequencing. Through the analysis of the obtained results, we found that the expression levels of most genes changed to varying degrees under the treatment of the two hormones, while the expression levels of a small portion of genes did not change significantly under the treatment of the hormones. Under the treatment of exogenous MeJA spray, the expression level of *MsCOI1-4/9/12* showed a significant up-regulation, while the gene expression level of *MsCOI1-3/14/19* showed a significant down-regulation trend (**Figure 6**). When observing the expression of COI1 family genes during exogenous ABA spraying treatment, we found that *MsCOI1-3/19* was significantly down-regulated, which was consistent with the trend during exogenous MeJA spraying. However, the expression level of *MsCOI1-8*, which showed no significant change during exogenous MeJA spraying, was significantly induced under ABA treatment.

### 3.8 Expression patterns of *MsCOI1* genes following AMV infection

**Figure 7:**
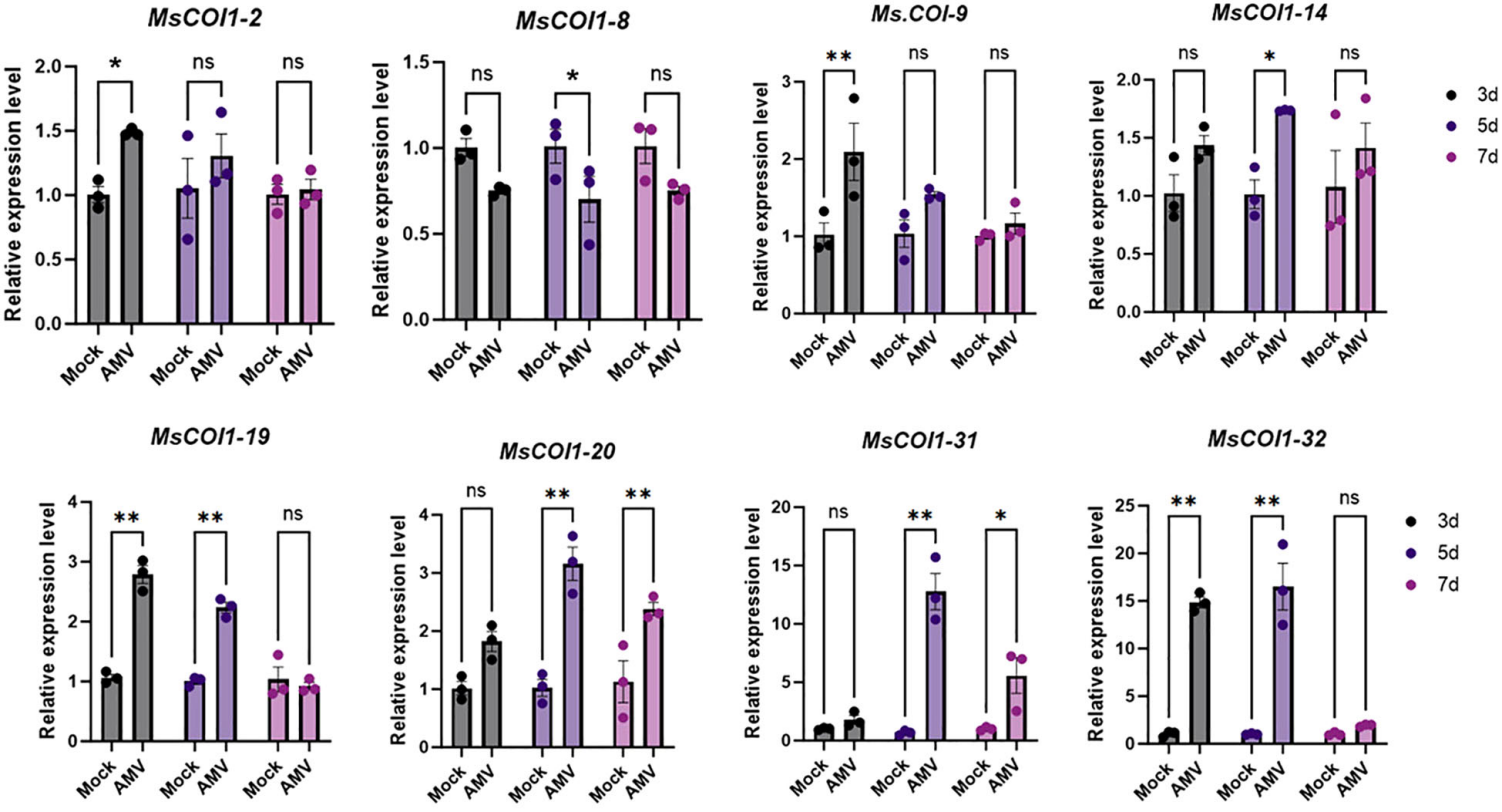
Expression analysis of eight *MsCOI1s* after AMV treatment. RT-qPCR analysis of the mRNA expression levels of *MsCOI1s* in the leaves of alfalfa plants at 3 dpi, 5 dpi, and 7 dpi after AMV treatment. The *MsActin* gene was used as the internal reference gene (**Supplementary Table 7**). Asterisks indicated significant differences [Student’s t-test, n=3, *p < 0.05, **p < 0.01; Error bars represent standard error (SE)]. “ns” indicates that there is no significant difference between the two sets of data.

To investigate the transcriptional responses of the *MsCOI1* gene family to AMV infection, eight *MsCOI1* genes containing defense and stress responsive cis-elements in their promoter regions were selected for quantitative Realtime PCR (RT-qPCR) validation at 3/5/7 days post-inoculation (dpi). Among these eight *MsCOI1* genes, six exhibited significant upregulation to varying degrees at distinct time points post-inoculation (**Figure 7**). Conversely, *MsCOI1–8* displayed significant downregulation specifically at 5 days post-inoculation. The *MsCOI1–19* gene showed no statistically significant alterations in transcript abundance throughout the 7 days infection period compared to mock-inoculated controls.

## 4 Discussion

This study delivers the first genome-wide identification and functional annotation of the *COI1* gene family in autotetraploid alfalfa (*Medicago sativa* L.), revealing 32 *MsCOI1* genes phylogenetically clustered into four subfamilies (I-IV). Critical structural features include a conserved three-exon architecture in 84.4% of genes and six essential motifs (Motif 1-6), with Motif 3 specifically absent in Subfamily IV-indicating potential subfunctionalization in JA signaling complex assembly. Promoter cis-element analysis unveiled pervasive stress responsive regulatory capacity: abscisic acid (ABRE, 93.8%) and MeJA (CGTCA/TGACG, 68.8%) elements dominate, while defense/stress-related elements (TC-rich repeats, 50%; MBS, 43.8%) further implicate *MsCOI1*s in abiotic and biotic adaptation. Matching the results of the promoter cis-acting element analysis is the expression of the *MsCOI1* family genes under hormone level treatment (**Figure 7**). *MsCOI1-4/9/12* was significantly induced by MeJA, and multiple MeJA response modules were noted in his promoter cis-acting element. Meanwhile, in the cis-acting element analysis of *MsCOI1-9*, there are some defense and stress responsiveness components. It suggests that it may act on plant defense by responding to the induction of the jasmonic acid signaling pathway. Expression profiling confirmed tissue specific patterns (*MsCOI1-24/25/26* in nodules/roots) and hormone inducibility (40.6% genes upregulated by MeJA/ABA), aligning with COI1’s canonical role as the JA receptor core within the ^SCF^COI1-JAZ-MYC2 module (Howe et al., 2018; Sheard et al., 2010). Meanwhile, *MsCOI1-24/25/26* are highly expressed in nodules/roots, which may suggest its function in the nitrogen fixation process of leguminous plants. Furthermore, as an autotetraploid genome plant, alfalfa presents a compelling case for in-depth investigation into the functional redundancy and specificity differentiation among the 32 *MsCOI1* genes identified in this study. By integrating our expression profile data, we can preliminarily distinguish genes playing a core role in AMV infection from members that are potentially functionally redundant. Specifically, four genes (*MsCOI1-19/20/31/32*; **Figure 6**) that were consistently upregulated upon AMV infection likely constitute a core response module in the jasmonic acid (JA) signaling pathway for early antiviral defense. This is further supported by the enrichment of MeJA-responsive elements in their promoter regions (**Figure 4**), reinforcing their central role in the JA pathway. Conversely, five genes (*MsCOI1-5/7/9/27/28*; **Figure 5**), which showed constitutively low expression across all tested tissues, may represent functionally redundant copies or genes activated only under specific conditions or developmental stages. Additionally, the three tightly linked *MsCOI1* genes on chromosome chr5.2 (**Figure 1**) potentially originated from recent tandem duplication events and may exhibit functional overlap among themselves. This systematic work addresses a critical gap in polyploid legumes, establishing a foundation for deploying *MsCOI1s* to combat AMV.

The landmark study by An et al. (2019) demonstrating that *ZmCOI1a* restores male fertility and fungal defense in *Arabidopsis* coi1–1 mutants underscores the dual functionality of COI1 across monocots and eudicots (An et al., 2019). Structural conservation is paramount: *ZmCOI1a’s* F-box domain mediates JAZ degradation via specific hydrogen bonding with JA-Ile (Sheard et al., 2010), while its LRR domain confers substrate specificity. In alfalfa, Subfamily I genes exhibit high homology to *AtCOI1* (Feys et al., 1994), and their nuclear localization (this study) mirrors *ZmCOI1a*’s nucleocytoplasmic shuttling for MYC2 release (An et al., 2019). Notably, Subfamily IV forms an alfalfa specific solitary branch without orthologs in *Arabidopsis*, rice, or maize (**Figure 2**), suggesting potential functional neofunctionalization in alfalfa. Similar uniqueness was only found in *Gossypium* COI1 (Li et al., 2022), it may reflect the sub-genomic functional differentiation in autotetraploid (Chen H. et al., 2020). This phylogenetic isolation implies unique adaptations-possibly novel JAZ-binding interfaces or pathogen effector targets-that diverge from canonical COI1 functions. To probe this, future work should resolve Subfamily IV protein structures via AlphaFold2 to identify divergent LRR domain epitope (Jumper et al., 2021). From another perspective we can conduct yeast-two-hybrid screening against JAZ repertoires to test binding specificity (Mao et al., 2025), and engineer domain-swap chimeras between Subfamily IV and *AtCOI1* to pinpoint functional determinants (Yousuke et al., 2018). Such approaches could unveil unprecedented JA signaling mechanisms.

AMV induced upregulation of six *MsCOI1*s (e.g., *MsCOI1-19, -20*) at 3–7 dpi signifies host attempts to activate JA defenses. This aligns with established antiviral mechanisms, such as JA driven ROS scavenging via *SlJAZ7* in tomato (Lin et al., 2024); JA systemic induce disease-resistant pathways (Steven et al., 2003) and resource reallocation to antiviral metabolites (Gareth, 2020). Conversely, *MsCOI1–8* significantly reduced at 5 dpi and there is a downward trend within 3 to 7 days after AMV vaccination. It exposes AMV’s likely counter strategy to suppress JA signaling a tactic employed by multiple viruses. For instance, CMV 2b protein directly stabilizes JAZ repressors by binding their ZIM domains, blocking COI1-JAZ interaction and MYC2 liberation (Wu et al., 2017). Similarly, TMV exploits JA-SA antagonism to attenuate host immunity (Oka et al., 2013). In alfalfa, promoter TC-rich repeats (defense/stress) in 50% and MeJA elements in 68.8% of *MsCOI1*s provide plausible targets for AMV effectors. The transient induction of *MsCOI1–14* by MeJA (**Figure 7**) and the activation of its expression during AMV infection (**Figure 6**) suggest that plants may resist viral infection through jasmonic acid-mediated defense responses. Despite these insights, tetraploid complexity poses functional redundancy challenges. Allelic variation among homologous chromosomes (e.g., four *MsCOI1*s on chr5.2) may buffer against loss of function mutations-necessitating CRISPR-Cas9 multiplex editing to dissect individual contributions (Yousuke et al., 2018). Furthermore, JA’s context dependent role in virus interactions requires resolution: Does AMV encode effectors that directly inhibit MsCOI1-JAZ binding, analogous to CMV 2b (Wu et al., 2017)? To leverage this, future work can validate COI1-JAZ protein interactions via co-immunoprecipitation in AMV infected plants and pyramid elite *MsCOI1* alleles using marker-assisted selection. Integrating these approaches will accelerate breeding of AMV resilient alfalfa, harnessing COI1’s dual roles to sustain productivity under pathogen pressure.

## 5 Conclusions

This study presents the first genome-wide identification and functional characterization of the *COI1* gene family in autotetraploid alfalfa (*Medicago sativa* L.), revealing 32 *MsCOI1* genes phylogenetically classified into four subfamilies (I-IV). Key structural characteristics of the *MsCOI1* family comprise a conserved three-exon organization across 84.4% of its members and six functionally critical motifs, with Motif 3 notably absent in Subfamily IV-an observation indicative of subfunctionalization in plant responses. Cis-element analysis demonstrated that 93.8% of promoters harbor abscisic acid responsive elements (ABRE), while 68.8% contain MeJA-responsive elements (CGTCA/TGACG), implicating *MsCOI1*s in hormonal crosstalk. Stress responsive elements (TC-rich repeats: 50%; MBS: 43.8%) further support roles in abiotic/biotic adaptation. Expression profiling confirmed tissue specificity (e.g., *MsCOI1-24/25/26* in nodules/roots) and hormone responsiveness (40.6% induced by MeJA/ABA). Crucially, six of eight tested *MsCOI1*s were upregulated during AMV infection (3–7 dpi), while *MsCOI1–8* was suppressed at 5 dpi, indicating JA pathway manipulation by the virus. These findings establish a foundation for leveraging *MsCOI1* genes to enhance viral resistance in alfalfa breeding programs.

## Data Availability

The datasets presented in this study can be found in online repositories. The names of the repository/repositories and accession number(s) can be found in the article/**Supplementary Material**.
